# MRI radiomics model for predicting TERT promoter mutation status in glioblastoma

**DOI:** 10.1002/brb3.3324

**Published:** 2023-12-06

**Authors:** Ling Chen, Runrong Chen, Tao Li, Lizhao Huang, Chuyun Tang, Yao Li, Zisan Zeng

**Affiliations:** ^1^ Department of Radiology The First Affiliated Hospital of Guangxi Medical University Nanning Guangxi China; ^2^ Department of Radiology Liuzhou Worker's Hospital The Fourth Affiliated Hospital Guangxi Medical University Nanning Guangxi China; ^3^ Department of Neurosurgery Liuzhou Worker's Hospital The Fourth Affiliated Hospital Guangxi Medical University Nanning Guangxi China

**Keywords:** glioblastoma, magnetic resonance imaging, radiomics, TERT

## Abstract

**Background and purpose:**

The presence of TERT promoter mutations has been associated with worse prognosis and resistance to therapy for patients with glioblastoma (GBM). This study aimed to determine whether the combination model of different feature selections and classification algorithms based on multiparameter MRI can be used to predict TERT subtype in GBM patients.

**Methods:**

A total of 143 patients were included in our retrospective study, and 2553 features were obtained. The datasets were randomly divided into training and test sets in a ratio of 7:3. The synthetic minority oversampling technique was used to achieve data balance. The Pearson correlation coefficients were used for dimension reduction. Three feature selections and five classification algorithms were used to model the selected features. Finally, 10‐fold cross validation was applied to the training dataset.

**Results:**

A model with eight features generated by recursive feature elimination (RFE) and linear discriminant analysis (LDA) showed the greatest diagnostic performance (area under the curve values for the training, validation, and testing sets: 0.983, 0.964, and 0.926, respectively), followed by relief and random forest (RF), analysis of variance and RF. Furthermore, the relief was the optimal feature selection for separately evaluating those five classification algorithms, and RF was the most preferable algorithm for separately assessing the three feature selectors. ADC entropy was the parameter that made the greatest contribution to the discrimination of TERT mutations.

**Conclusions:**

Radiomics model generated by RFE and LDA mainly based on ADC entropy showed good performance in predicting TERT promoter mutations in GBM.

## Graphical abstract

Adult glioblastoma (GBM) is the most common and aggressive primary brain tumor, with a high recurrence and mortality rate. The presence of TERT promoter mutations has been associated with poor prognosis and resistance to therapy, making it an important biomarker for personalized treatment strategies. In this study, we investigated the relationship between multiparameter MRI features and TERT mutation status, establishing a radiomics signature for predicting TERT promoter mutation status, and verified its effectiveness in prognostic assessment with GBM patients.

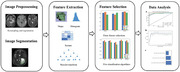

## 1 INTRODUCTION

Glioblastoma (GBM) is the most common and aggressive primary brain tumor in adults (Chougule et al., 2022; Gonçalves et al., 2020), with high recurrence and mortality rates despite standard therapies (Campos et al., 2016; Parvaze et al., 2023). Molecular classification of GBM has been proposed to identify subtypes with distinct clinical, genetic, and epigenetic features for risk stratification (Gritsch et al., 2022; Yang et al., 2022). TERT promoter mutations, one of the most important molecular biomarkers, are present in up to 73.6% of GBM cases (Kanas et al., 2017). The presence of TERT promoter mutations is associated with worse prognosis and resistance to therapy, making it an important biomarker for personalized treatment strategies (Śledzińska et al., 2021).

TERT is an enzyme that is essential for the maintenance of telomeres, the protective caps on the ends of chromosomes (Arita, Narita et al., 2013; Stichel et al., 2018). Telomeres shorten with each cell division, eventually leading to cellular senescence. TERT adds DNA to the ends of telomeres, preventing them from shortening and allowing the cell to continue dividing (Amen et al., 2021). Usually, TERT is highly expressed in stem cells and cancer cells. TERT mutation is a hallmark of cancer and is often used as a diagnostic and prognostic marker. In GBM, TERT promoter has been shown to be an independent prognostic factor for poor overall survival (OS) and progression‐free survival (PFS), even after adjusting for age, grade, and other molecular markers. Previous research has demonstrated that suppressing TERT expression increases the sensitivity of cell to radiation‐ and chemotherapy‐induced DNA damage, making it a target for novel therapeutic approaches (Nakamura et al., 2005; Rohwer et al., 2013).

The ability to identify TERT mutations is essential for the risk stratification of GBM patients in clinical settings. Molecular diagnostic procedures like polymerase chain reaction or next‐generation sequencing are frequently used to detect TERT promoter mutations (Fujioka et al., 2021; Jovčevska, 2018). However, the acquisition of pathology specimens is challenging in clinical settings where surgery is not possible or cannot be tolerated by the patient. Furthermore, there is always the possibility of false‐positive or false‐negative results. To overcome these limitations, it is important to integrate multiple diagnostic technologies to increase the diagnostic efficiency and accuracy.

Recently, radiomics is being increasingly used as a supplementary tool for tumor diagnoses and the monitoring of therapeutic response (Li, Li et al., 2022; Li, Liu et al., 2022). Radiomics involves the extraction of quantitative features from medical images, which can then be used to identify patterns and relationships within the data (Jiang et al., 2017). This approach can help identify subtle changes in tumor characteristics that may be missed by traditional imaging techniques. However, some traditional radiomics approaches tend to use a single algorithm to build models, which may not be able to adequately represent the complexity and heterogeneity of the imaging data. Therefore, further research is required to apply a variety of algorithms along with feature selections in order to increase the accuracy of the models.

To the best of our knowledge, two studies have been conducted on the radiomics analysis of TERT classification in GBM (Gerardi et al., 2023; Zhang et al., 2023); however, these researchers do not encompass the selection of optimal algorithms and feature selectors for predicting TERT classification. Despite the valuable insights gained from these two studies, there are still several limitations that need to be addressed. The studies have not examined the potential impact of combining different feature selections and algorithms on the accuracy of TERT classification. Integrating multiple sources of information could potentially lead to more precise and reliable predictions, but this hypothesis has not been thoroughly tested. Based on the reasons outlined above, we investigated the radiomics model for distinguishing TERT promoter mutations based on preoperative multiparameter MRI by applying three feature selections and five classification algorithms.

## 2 METHODS

### 2.1 Patient selection

**FIGURE 1:**
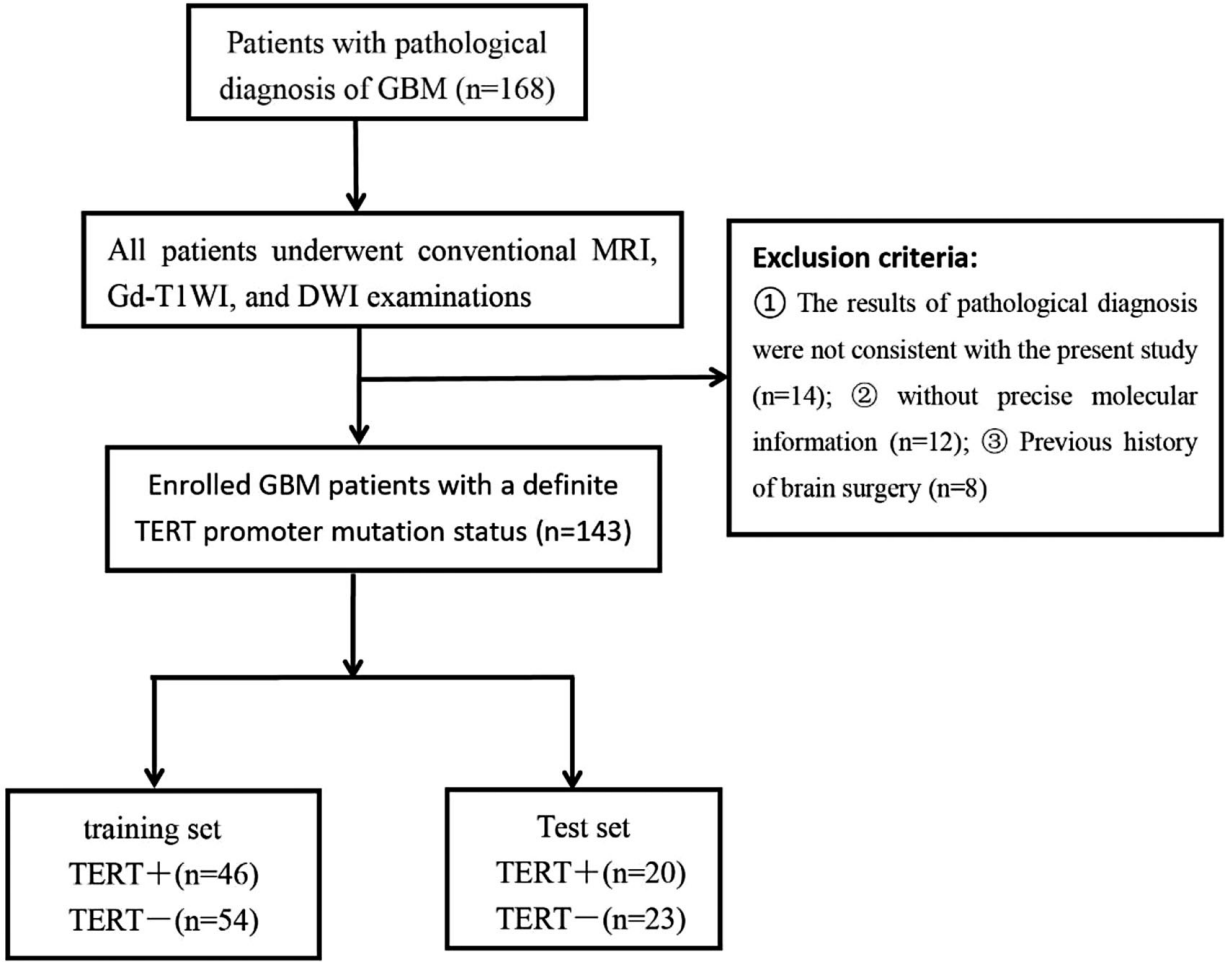
The patient selection flow chart.

This retrospective study was approved by the institutional research ethics review board, and the requirement for obtaining patient consent was waived. One hundred and forty‐three GBM patients with a confirmed molecular diagnosis of TERT were included in our cohort between January 2019 and December 2022. Clinical information, such as age, sex, and genetic diagnosis results (including IDH and TERT), were collected from hospital information system. The MRI images were obtained from the archiving and communication systems. The inclusion criteria were as follows: (1) pathological diagnosis consistent with the study; (2) time elapsed between MRI examination and surgery not exceeding 1 week; (3) no history of surgery or chemoradiotherapy; (4) patients were not on steroids at the time of imaging. The patient selection flow chart is shown in Figure 1.

### 2.2 MRI protocol

Imaging data included axial T2‐weighted, DWI, and ADC sequences obtained on a 1.5T (GE, Octane; Siemens) or 3.0T MRI system (Philips, Achieva; GE, Premier). The MRI parameters are provided in Table 1.

**TABLE 1:** Magnetic resonance imaging protocol.

	3.0 T Philips	1.5 T Siemens	1.5 T GE	3.0 T GE
**T2WI**				
TR/TE (ms)	2600/80	4950/104	4460/126.2	5844/129
FOV	512 × 512	378 × 448	512 × 512	512 × 512
FA (°)	90	90	90	90
Matrix	256 × 217	256 × 203	512 × 192	256 × 217
Slice thickness/gap (mm)	5/3	5/2.5	5/2.5	5/2.5
**DWI**				
TR/TE (ms)	2429/81	4850/81	5500/99.4	2155/61.2
FOV	192 × 192	196 × 196	256 × 256	256 × 256
FA (°)	90	90	90	90
Matrix	120 × 127	256 × 203	128 × 128	256 × 217
Slice thickness/gap (mm)	5/1	5/1	5/2.5	5/1

### 2.3 Pathological assessment

Pathological diagnosis was made according to the 2021 (fifth edition) classification criteria for central nervous system brain tumors. IDH and TERT mutation statuses were obtained by next‐generation sequencing, as described elsewhere (Arita, Narita, Takami et al., 2013; Hasanau et al., 2022).

### 2.4 Radiomics process

#### 2.4.1 Image preprocessing and segmentation

**FIGURE 2:**
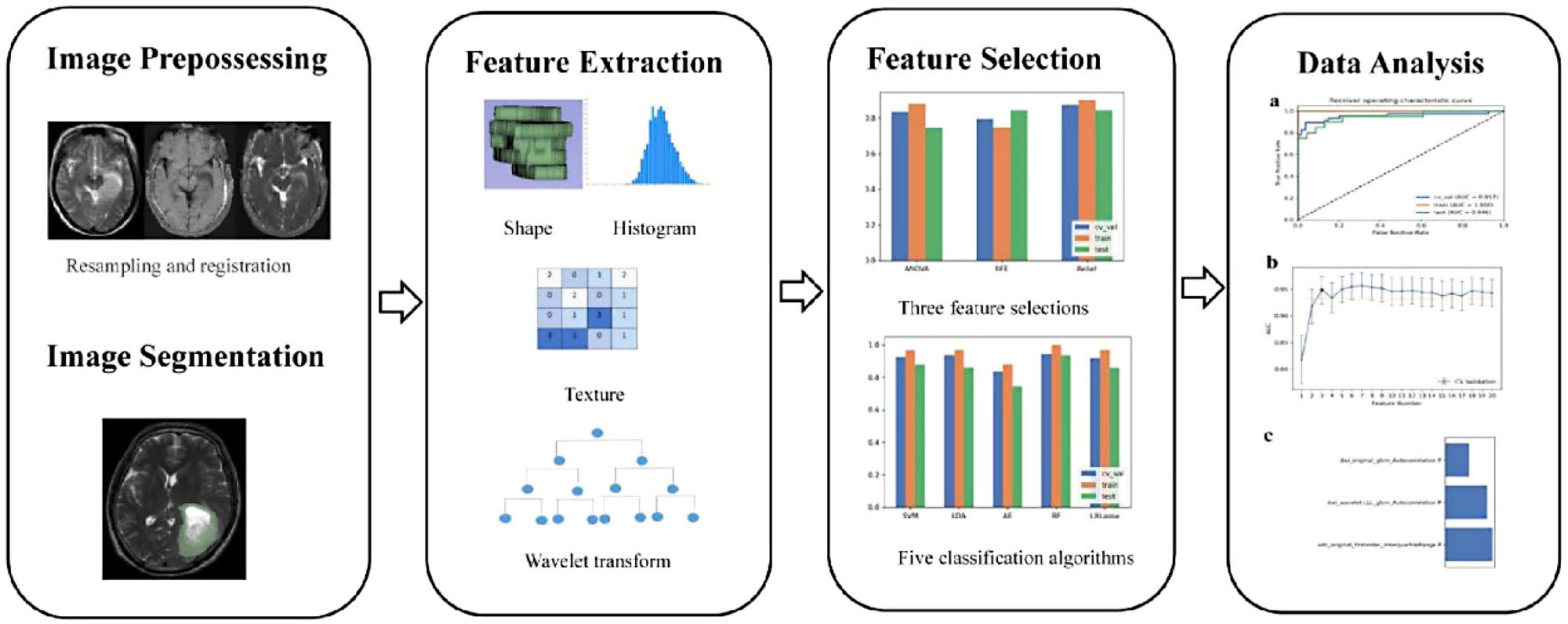
Radiomics processing flow.

The DICOM format images of T2WI, DWI, and ADC were imported into 3D slicer software (version 5.3.0; https://www.slicer.org/). The images were resampled to a voxel size of 1 mm × 1 mm × 1 mm, and the gray level was discretized with a bin width of 25. These steps helped reduce the variability caused by differences in scanning parameters and equipment. The volume of interest was semiautomatically plotted on T2WI along the tumor margin slice by slice and automatically registered to DWI and ADC images. Tumor segmentation was manually performed by two neuroradiologists (T.L and L.Z.H) with 10 years of experience in neuroradiology. Interclass correlation coefficient between 0.75 and 1 was considered indicative of good agreement. Any disagreement between the two neuroradiologists was resolved by consensus. The radiomics process is shown in Figure 2.

#### 2.4.2 Feature extraction

For each patient, a total of 2553 features were extracted. These features can be broadly categorized into four groups, including shape features (*n* = 14), histogram features (*n* = 18), textural features (gray‐level co‐occurrence matrix [*n* = 24], gray‐level dependence matrix [*n* = 14], gray‐level run length matrix [*n* = 16], gray‐level size zone matrix [*n* = 16], neighborhood gray‐tone difference matrix [*n* = 5]), and wavelet transform (*n* = 744). The texture features of the above segmentation images were extracted and quantified in the pyradiomics database after wavelet transformation. Each stage of wavelet filtering results in eight decompositions. In three dimensions, all feasible combinations of high‐pass or low‐pass filters (LLH, LHL, LHH, HLL, HLH, HHL, HHH, LLL) were applied, and three types of texture features were retrieved at each decomposition. Finally, the wavelet transform yielded 744 features, and a total of 851 texture features were retrieved per sequence. The feature classification and radiomics parameters are shown in Table 2.

**TABLE 2:** The classification and parameter composition of radiomics features.

Feature classifier	Feature parameters (*n* = 851)
Shape feature (*n* = 14)	Elongation, flatness, least axis length, major axis length, maximum 2D diameter column, maximum 2D diameter row, maximum 2D diameter slice, maximum 3D diameter, mesh volume, minor axis length, sphericity, surface area, surface volume ratio, voxel volume
Histogram feature (*n* = 18)	P10, P90, interquartile range, energy, entropy, skewness, kurtosis, maximum, minimum, mean, mean absolute, deviation, median, total energy, uniformity, variance, range, robust mean absolute deviation, root mean squared
Texture feature (*n* = 75)	Gray‐level co‐occurrence matrix, GLCM (*n* = 24); gray‐level dependence matrix, GLDM (*n* = 14); gray‐level run length matrix, GLRLM (*n* = 16); gray‐level size zone matrix, GLSZM (*n* = 16); neighborhood gray‐tone difference matrix, NGTDM (*n* = 5)
Wavelet transform (*n* = 744)	Wavelet filtering produces eight decompositions per stage. In the three dimensions, all feasible combinations of high‐pass or low‐pass filters (LLH, LHL, LHH, HLL, HLH, HHL, HHH, LLL)

#### 2.4.3 Feature selection and model exploration

All processes were implemented with FeAture Explorer (FAE) (https://github.com/salan668/version 0.3.7) on Python (3.7.6). The pipelines were built based on scikit‐learn (version 0.22.2). The datasets were randomly divided into two groups (the training set and the test set) in a ratio of 7:3. To remove the imbalance of the training dataset, we used the synthetic minority oversampling technique to make the samples balanced. On the feature matrix, we computed the mean value and the standard deviation for each feature vector. Each feature vector was split by the standard deviation, and its mean value was subtracted. Each vector has a zero center and a unit standard deviation after normalization. We compared the similarity of each feature pair because of the large dimension of the feature space. One of the feature pairs was eliminated if the Pearson correlation coefficient value was more than 0.99. Using this process, the dimension of feature space is reduced, rendering each feature independent of each other.

Analysis of variance (ANOVA), recursive feature elimination (RFE), and relief were the three feature selections employed before building the model to select the features that showed the strongest correlation to the label (TERT mutations). To assess the association between the features and the label, the *F*‐value was determined. To develop the model, we chose a specific amount of features and ranked them according to the matching *F*‐value. Then, we filtered the features based on their *F*‐values and built the model with a specific amount of features. The selected features were then modeled using support vector machine (SVM), auto encoder (AE), linear discriminant analysis (LDA), random forest (RF), and logistic regression via the least absolute shrinkage and selection operator (LR‐Lasso) classification methods. Finally, we used 10‐fold cross validation on the training dataset to establish the model's hyperparameters. The hyperparameters were chosen based on the performance of the model on the validation dataset.

The performance of the model was evaluated using receiver‐operating characteristic (ROC) curve analysis, and the area under the curve (AUC) values were calculated for quantification. At a cutoff value associated with the maximum Youden index, the accuracy, sensitivity, specificity, positive predictive value, and negative predictive value were also determined. We also estimated the 95% confidence interval by bootstrapping with 1000 samples.

#### 2.4.4 Statistical analysis

SPSS 27.0 was used for statistical analyses. The age was compared using the independent‐samples *t* test, and sex distribution was compared using the chi‐square test. The diagnostic accuracy in the training set, validation set, and test set was evaluated by ROC curve analysis. *p* Values <.05 were considered indicative of statistical significance.

## 3 RESULTS

### 3.1 Basic clinical information of participants

The basic clinical characteristics of the GBM patients are shown in Table 3. One hundred and forty‐three patients (58 females, 85 males; mean age, 52.68 ± 11.02 years [range, 27–74]) were enrolled in this study. Of these, 14 cases were excluded as the results of pathological diagnosis were not consistent with the present study (metastatic tumor, *n* = 6; meningioma, *n* = 8), 7 cases were excluded because of the lack of precise molecular information, and 4 cases were omitted due to a previous history of brain surgery. Sixty‐six cases (46.15%) were diagnosed as TERT mutation‐positive and 77 (53.85%) as TERT mutation‐negative. There were significant differences in age among the TERT subgroups. However, the sex distribution did not show a significant difference in the TERT mutation subgroups (*p* = .03).

**TABLE 3:** The basic clinical characteristics of the glioblastoma (GBM) patients.

	Training set (*n* = 100)			Test set (*n* = 43)			
Characteristics	TERT‐mt	TERT‐wt	*p*	TERT‐mt	TERT‐wt	*p*	*p*
**Age (mean ± SD)**	57.34 ± 11.03	53.26 ± 8.57	0.032	56.44 ± 10.38	53.08 ± 9.34	0.028	0.569
**Male**	25	32		13	17		
**Sex**			0.169			0.011	0.962
**Female**	21	22		7	6		
**TERT**	46	54	0.642	20	23	0.371	0.058

Abbreviations: TERT‐mt, telomerase reverse transcriptase mutant‐type; TERT‐wt, telomerase reverse transcriptase wild‐type.

### 3.2 Radiomics results

We compared all models using FAE on the validation set. The results of ROC curve analysis for three feature selections and five classification algorithms with fivefold cross validation are shown in Table 4.

**TABLE 4:** Results of receiver‐operating characteristic (ROC) curve analysis for various feature selections and classification algorithms with 10‐fold cross validation.

Feature set	AUC	95% CI	YI	Acc	Sen	Spe	PPV	NPV
Zscore_PCC_ANOVA_6_LRLasso	0.936	[0.875–0.987]	0.567	0.940	0.891	0.982	0.976	0.914
Zscore_PCC_ANOVA_7_SVM	0.938	[0.882–0.983]	0.546	0.920	0.870	0.963	0.952	0.897
Zscore_PCC_ANOVA_7_AE	0.928	[0.852–0.976]	0.576	0.90	0.848	0.944	0.929	0.879
Zscore_PCC_ANOVA_7_RF	0.957	[0.898–0.992]	0.575	0.930	0.891	0.963	0.954	0.912
Zscore_PCC_ANOVA_9_LDA	0.942	[0.884–0.985]	0.602	0.940	0.870	1	1	0.900
Zscore_PCC_RFE_7_AE	0.940	[0.879–0.983]	0.546	0.920	0.891	0.944	0.932	0.911
Zscore_PCC_RFE_9_SVM	0.964	[0.926–0.992]	0.455	0.930	0.891	0.963	0.9534	0.912
Zscore_PCC_RFE_9_LDA	0.964	[0.924–0.993]	0.462	0.940	0.891	0.982	0.976	0.914
Zscore_PCC_RFE_9_LRLasso	0.955	[0.913–0.988]	0.387	0.930	0.891	0.963	0.954	0.912
Zscore_PCC_RFE_12_RF	0.959	[0.905–0.995]	0.475	0.930	0.891	0.963	0.954	0.912
Zscore_PCC_Relief_7_AE	0.938	[0.883–0.981]	0.583	0.900	0.848	0.944	0.929	0.879
Zscore_PCC_Relief_8_SVM	0.9384	[0.883–0.985]	0.572	0.920	0.870	0.963	0.952	0.897
Zscore_PCC_Relief_11_LDA	0.940	[0.886–0.985]	0.641	0.940	0.870	1	1	0.900
Zscore_PCC_Relief_13_LRLasso	0.942	[0.887–0.984]	0.795	0.920	0.826	1	1	0.871
Zscore_PCC_Relief_14_RF	0.961	[0.911–0.991]	0.550	0.930	0.891	0.963	0.954	0.912

Abbreviations: ROC, receiver‐operating characteristic; AUC, area under the curve; YI, Youden index; Acc, accuracy; Sen, sensitivity; Spe, specificity; PPV, positive predictive value; NPV, negative predictive value.

**FIGURE 3:**
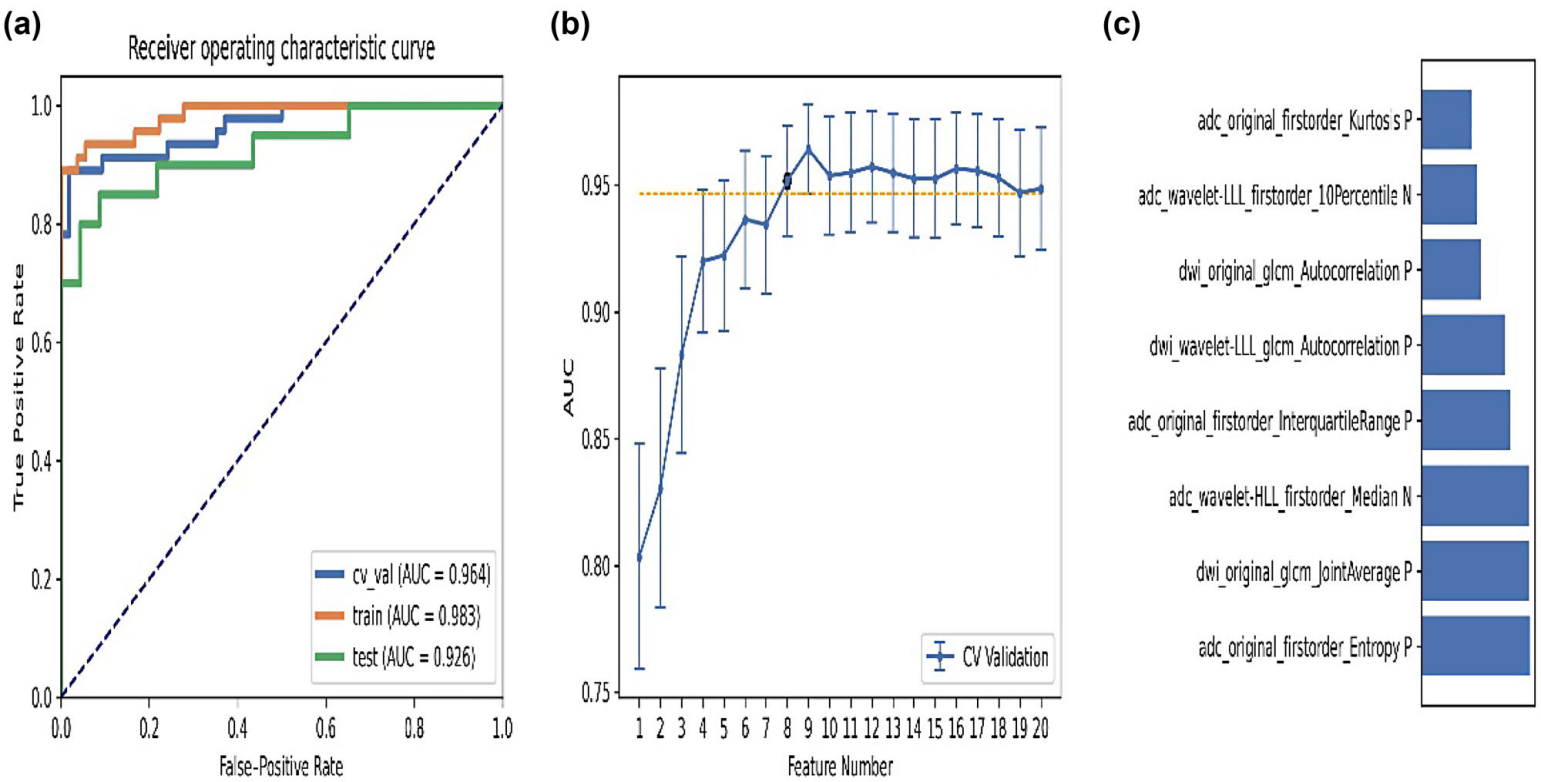
Model performance generated by recursive feature elimination (RFE) and linear discriminant analysis (LDA): (**a**) receiver‐operating characteristic (ROC) curves of different datasets; (**b**) a simpler model of eight features based on the “one standard error” principle; (**c**) feature contribution arrangement in the final model generated by RFE and LDA.

The pipeline based on RFE and LDA generated the highest AUC with nine features. When using the “one standard error” rule (Wang et al., 2022), FAE produces a simpler model of eight features. The AUC of the cross verification set, training set, and test set were 0.964, 0.983, and 0.926, respectively. The eight features selected from this pipeline were listed in ascending order, as shown in Figure 3. The result demonstrated that the feature that contributed most to the whole pipeline was the entropy of the original first‐order feature from ADC.

**FIGURE 4:**
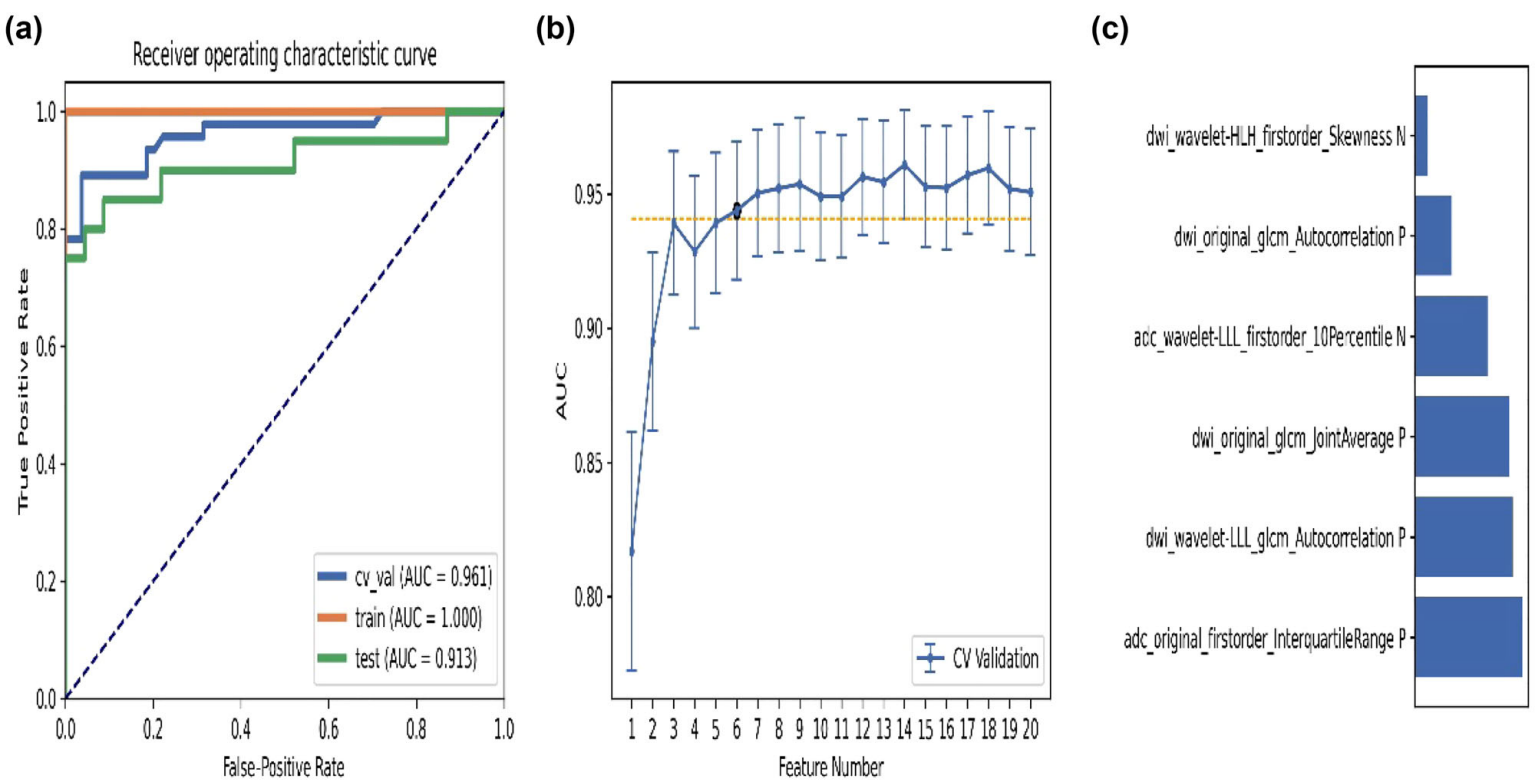
Model performance generated by relief and random forest (RF): (**a**) receiver‐operating characteristic (ROC) curves of different datasets; (**b**) a simpler model of six features based on the “one standard error” principle; (**c**) feature contribution arrangement in the final model generated by relief and RF.

The pipeline combined with relief and RF produced the highest AUC of 14 features, and the “one standard error” rule was used to screen out the 6 features. The AUC of the cross verification set, training set, and test set were 0.961, 1.0, and 0.913, respectively. The six features selected from this pipeline were listed in ascending order, as shown in Figure 4. The result showed that the feature that contributed most to the whole pipeline was the interquartile range of the original first‐order from ADC.

**FIGURE 5:**
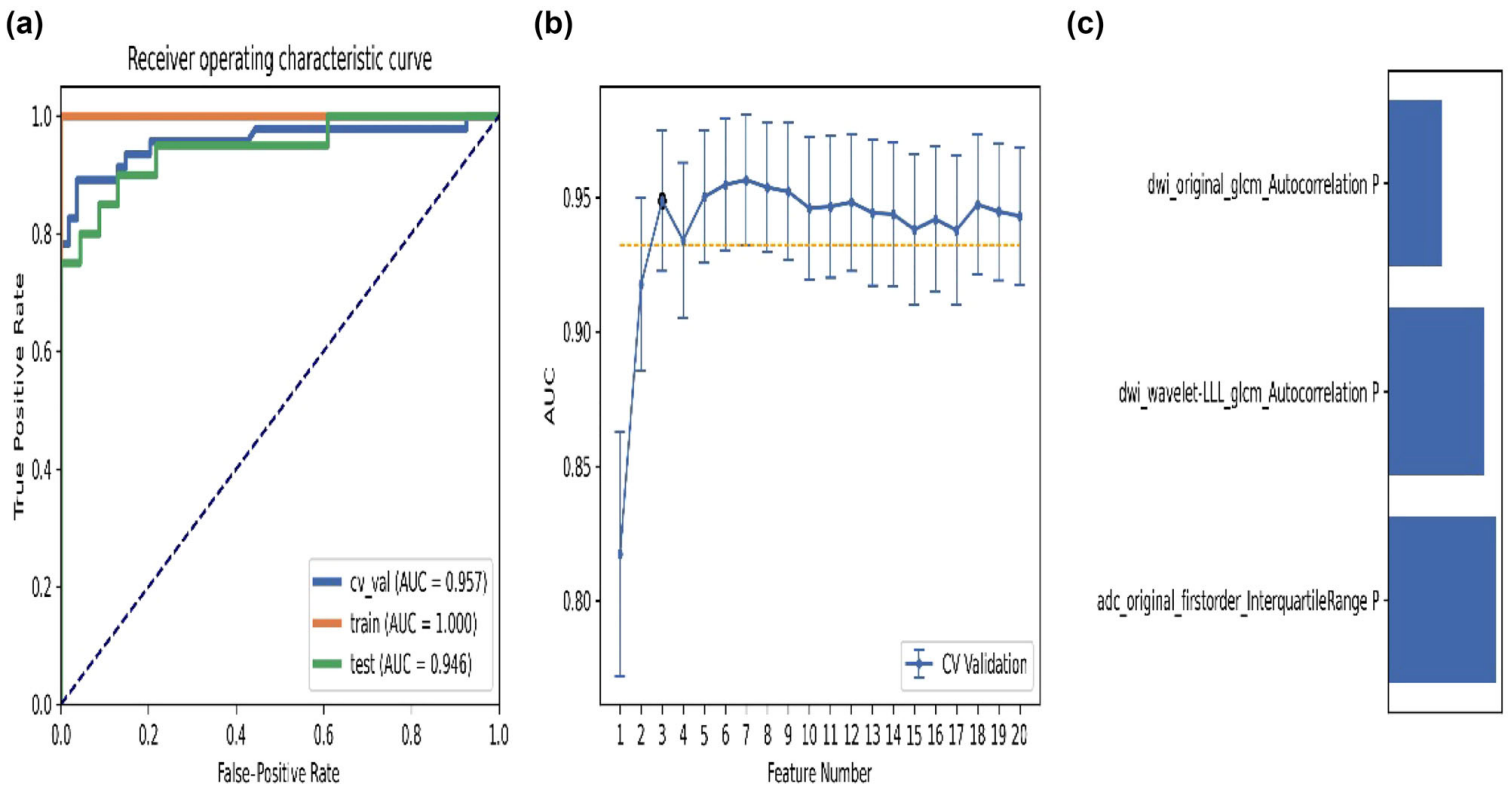
Model performance generated by analysis of variance (ANOVA) and random forest (RF): (**a**) receiver‐operating characteristic (ROC) curves of different datasets; (**b**) a simpler model of three features based on the “one standard error” principle; (**c**) feature contribution arrangement in the final model generated by ANOVA and RF.

The pipeline coupled with ANOVA and RF produced the highest AUC of seven features, and the “one standard error” rule was used to screen out a simpler model with three features. The AUC values of the cross verification set, training set, and test set were 0.957, 1.0, and 0.946, respectively. The three features selected from this pipeline were listed in ascending order, as shown in Figure 5. The result demonstrated that the feature that contributed most to the whole pipeline was the interquartile range of the original first‐order from ADC.

**FIGURE 6:**
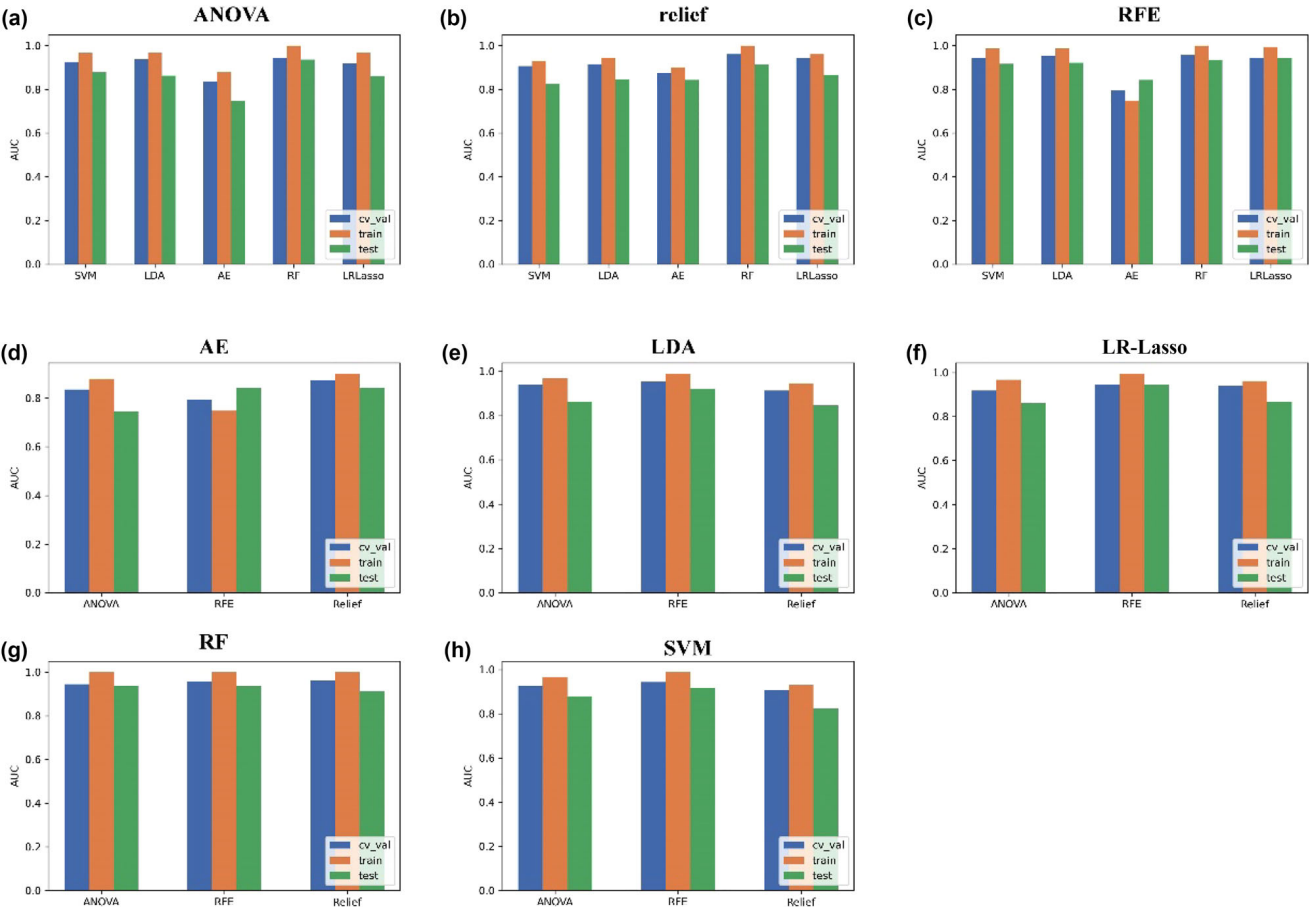
The comparison of different feature selectors and classification algorithms. Figure a‐c showed that the ANOVA, relief, and RFE feature selectors were combined with these five algorithms to evaluate the model performance. Figure d‐h showed that AE, LDA, LR‐Lasso, RF, SVM algorithms were integrated with these three feature selectors to evaluate the diagnostic efficiency of the model.

The comparison of different feature selectors and classification algorithms is shown in Figure 6a–h. The model evaluated by relief demonstrated remarkable stability with AUC values exceeding 0.800 on the validation, training, and test sets when evaluating the classification algorithms (SVM, LDA, AE, RF, and LR‐Lasso) individually in conjunction with these three feature selectors (ANOVA, RFE, and relief). However, employing the Delong test for statistical analysis revealed no significant differences in distinguishing TERT subtypes in patients with GBM between the combination of relief and the other five classification algorithms. The performance of five classification algorithms was evaluated using ANOVA, RFE, and relief feature selectors. Among them, RF demonstrated the highest stability and achieved AUC values exceeding 0.913. Furthermore, the Delong test indicated no significant differences in distinguishing TERT subtypes in patients with GBM when comparing RF with the other three feature selectors. When five classification algorithms were performed to access ANOVA, RFE, and relief feature selectors, RF was the most stable, and the AUC values were higher than 0.913. The Delong test revealed that RF combined with the other three feature selectors did not make a significant difference in distinguishing TERT subtypes in GBM patients.

## 4 DISCUSSION

This study explored the potential value of radiomic models based on T2WI, DWI, and ADC maps using different feature selection (ANOVA, RFE, and relief) and classification algorithms (SVM, LDA, AE, RF, and LR‐Lasso) to predict the presence of TERT promoter mutations in patients with GBM. Our cohort of 143 GBM patients comprised 46.15% tumors with TERT mutation. The age distribution among the TERT subgroups exhibited significant disparities; however, no statistically significant variation was observed in the sex distribution across the TERT mutation subgroups. Furthermore, using feature selectors to separately evaluate other five classification algorithms, relief was considered the best feature selector with AUC values more than 0.800 across validation set, training set, and test set. On the other hand, RF was found to be the preferred classifier for separately evaluating the performance of these three feature selectors, and the AUC values were greater than 0.913. In our research, different combinations of feature selectors and classification algorithms generated different results in predicting the TERT mutation. As for combination, the model constructed based on the RFE and LDA yielded the best diagnostic performance (AUC, accuracy, sensitivity, and specificity: 0.964, 0.940, 0.891, and 0.982, respectively) compared to other combinations.

Some previous studies focused on a single radiomic approach, and the diagnostic efficacy of these models was far from satisfactory. In a retrospective study of 112 patients with newly diagnosed GBM by Yamashita et al. (2019), the AUC, accuracy, sensitivity, and specificity of the radiomics model using SVM for predicting TERT promoter mutation were 0.776, 0.857, 0.548, and 0.741, respectively. In another study of 105 gliomas by Peng et al. (2021), the AUC and accuracy of classifying IDH status by the Lasso method were 0.770 and 0.823. In general, the performance of the model increases with the size of the dataset (Rogers et al., 2022). However, even in a large cohort, the classification results obtained with a single algorithm are still unsatisfactory. Yan et al. investigated 357 glioma patients by using Bayesian neural networks and integrated radiomics features from Gd‐T1 and ADC; the AUC, accuracy, sensitivity, and specificity for predicting TERT mutations were 0.598, 0.685, 0.976, and 0.290, respectively (Rogers et al., 2022). In this regard, it is very important to use appropriate algorithms to build the effectiveness model.

Our hypothesis is further verified by comparing the model performance generated by different feature selectors and classification algorithms (Chen et al., 2022; Zhang et al., 2023). A previous study sought to distinguish between 36 low‐grade gliomas and 42 GBMs using 4 machine learning classifiers (including SVM; *K*‐nearest neighbors, K‐NN; LDA; and adaptive boosting using decision stumps as the base learner, AdaBoost); the results showed that the AdaBoost classifier generated higher predictive accuracy than other algorithms individually (the AUC, sensitivity, specificity, and accuracy were 0.96, 91%, 86%, and 89%, respectively) (Malik et al., 2021). Zhang et al. (2023) developed a deep learning signature and various convolutional neural network (CNN) algorithms; the result revealed that ResNet50 had a high accuracy in predicting TERT mutation status (AUC: 0.890 and 0.990 in validation set and training set, respectively). Similarly, Sekhar et al. (2022) reported more accurate classification of brain cancers using SVM algorithm and fine‐tuned GoogLeNet features compared to discrete wavelet transform and CNN (accuracy: 100%, 98.7%, and 96.97%, respectively). The above‐cited studies demonstrate the potential for multiple algorithmic models to enhance their overall effectiveness when used in combination.

Several radiomics models have been developed in previous research to predict TERT promoter mutation status in patients with gliomas. However, the majority of previous studies have primarily focused on grades 2–4 gliomas. Limited clinical research has been conducted utilizing more feature selection techniques (such as ANOVA, RFE, and relief) and classification algorithms (including SVM, AE, LDA, RF, and LR‐Lasso) specifically for TERT subtypes in GBM. Our findings suggest that the model constructed based on the combination of RFE and LDA demonstrated superior diagnostic performance compared to other combinations. Previous studies have examined variations in perfusion metrics, magnetic resonance spectroscopy, T1 pre‐ and postcontrast, and T2WI‐FLAIR in TERT‐mutated GBMs harboring TERT mutations, yielding promising results (Calabrese et al., 2022; Tian et al., 2020; Yan et al., 2021). In this study, we aimed to compare the effectiveness of ADC, DWI, and T2WI in differentiating TERT mutation status in GBM patients using radiomics analysis. Our study indicated that ADC was the most significant parameter for the predictive model. This result is consistent with previous studies (Gihr et al., 2020; Park et al., 2021). Furthermore, among the radiomics features analyzed in this study, ADC entropy was found to have made the greatest contribution to the discrimination between TERT‐mutant and wild‐type GBMs. Specifically, the entropy feature was able to capture the heterogeneity of tumor microenvironment, which is a crucial factor in the development and progression of GBM and showed a strong correlation with TERT mutations (Gihr et al., 2022; Kim et al., 2020; Wang et al., 2023). Entropy is a measure of the randomness or disorder in the pixel intensity distribution of an image (Just, 2014). Theoretically, the greater the entropy, the greater is the dispersion degree of the tumor. In addition, entropy values were also significantly higher in TERT mutations, indicating increased heterogeneity within the tumor environment. The presence of high entropy values in TERT‐mutant GBM may be attributed to the dysregulated cellular metabolism, which results in differences in tumor microenvironment between TERT‐mutant and wild‐type tumors. From this perspective, entropy may serve as a useful marker and provide insights into the complex nature of tumor heterogeneity. Further research is required to validate these findings in larger cohorts and to investigate the clinical implications of using more imaging biomarkers in the diagnosis and treatment of GBM.

Some limitations of our study need to be considered. To begin, we need to expand the sample size of our dataset in order to draw more robust conclusions. Future study should incorporate samples from more centers. Second, in our investigation, only conventional sequences were employed, and subsequent analysis was carried out utilizing more advanced imaging techniques, such as perfusion imaging and amide proton transfer weighted imaging, which are sensitive to tumor heterogeneity. Last, we did not assess OS or PFS in GBM patients. Our future research will entail more in‐depth analysis incorporating the clinical characteristics.

In conclusion, our study highlights the potential value of ADC entropy as a potential noninvasive imaging biomarker for identifying TERT status in patients with GBM. The combination of feature selectors and classification algorithms has an important impact on predicting TERT mutations in GBM. The model obtained by RFE and LDA showed the best predictive value, which is of great significance for the development of more personalized therapeutic strategies in clinical settings.

## AUTHOR CONTRIBUTIONS

**Ling Chen**: Funding acquisition; writing—original draft; writing—review and editing. **Runrong Chen**: Data curation; formal analysis; resources; software. **Tao Li**: Funding acquisition; investigation; methodology; project administration. **Lizhao Huang**: Software; supervision; validation. **Chuyun Tang**: Investigation; methodology; project administration; visualization. **Yao Li**: Investigation; resources; supervision. **Zisan Zeng**: Conceptualization; project administration; supervision.

## CONFLICT OF INTEREST STATEMENT

The authors declare that they have no known conflicts of interest or personal relationships that could have appeared to influence the work reported in this paper.

### PEER REVIEW

The peer review history for this article is available at https://publons.com/publon/10.1002/brb3.3324.

## Data Availability

The data that support the findings of this study are available from the corresponding author upon reasonable request.

## References

[brb33324-bib-0001] Amen, A. M. , Fellmann, C. , Soczek, K. M. , Ren, S. M. , Lew, R. J. , Knott, G. J. , Park, J. E. , Mckinney, A. M. , Mancini, A. , Doudna, J. A. , & Costello, J. F. (2021). Cancer‐specific loss of TERT activation sensitizes glioblastoma to DNA damage. PNAS, 118, e2008772118. 10.1073/pnas.2008772118 33758097 PMC8020668

[brb33324-bib-0002] Arita, H. , Narita, Y. , Fukushima, S. , Tateishi, K. , Matsushita, Y. , Yoshida, A. , Miyakita, Y. , Ohno, M. , Collins, V. P. , Kawahara, N. , Shibui, S. , & Ichimura, K. (2013). Upregulating mutations in the TERT promoter commonly occur in adult malignant gliomas and are strongly associated with total 1p19q loss. Acta Neuropathologica, 126, 267–276. 10.1007/s00401-013-1141-6 23764841

[brb33324-bib-0003] Arita, H. , Narita, Y. , Takami, H. , Fukushima, S. , Matsushita, Y. , Yoshida, A. , Miyakita, Y. , Ohno, M. , Shibui, S. , & Ichimura, K. (2013). TERT promoter mutations rather than methylation are the main mechanism for TERT upregulation in adult gliomas. Acta Neuropathologica, 126, 939–941. 10.1007/s00401-013-1203-9 24174165

[brb33324-bib-0004] Calabrese, E. , Rudie, J. D. , Rauschecker, A. M. , Villanueva‐Meyer, J. E. , Clarke, J. L. , Solomon, D. A. , & Cha, S. (2022). Combining radiomics and deep convolutional neural network features from preoperative MRI for predicting clinically relevant genetic biomarkers in glioblastoma. Neurooncology Advances, 4, vdac060.. 10.1093/noajnl/vdac060 PMC9122791 35611269

[brb33324-bib-0005] Campos, B. , Olsen, L. R. , Urup, T. , & Poulsen, H. S. (2016). A comprehensive profile of recurrent glioblastoma. Oncogene, 35, 5819–5825. 10.1038/onc.2016.85 27041580

[brb33324-bib-0006] Chen, C. , Qin, Y. , Chen, H. , Cheng, J. , He, B. , Wan, Y. , Zhu, D. , Gao, F. , & Zhou, X. (2022). Machine learning to differentiate small round cell malignant tumors and non‐small round cell malignant tumors of the nasal and paranasal sinuses using apparent diffusion coefficient values. European Radiology, 32, 3819–3829. 10.1007/s00330-021-08465-w 35029732 PMC9123077

[brb33324-bib-0007] Chougule, T. , Gupta, R. K. , Saini, J. , Agrawal, S. , Gupta, M. , Vakharia, N. , Singh, A. , Patir, R. , Vaishya, S. , & Ingalhalikar, M. (2022). Radiomics signature for temporal evolution and recurrence patterns of glioblastoma using multimodal magnetic resonance imaging. NMR in Biomedicine, 35, e4647. 10.1002/nbm.4647 34766380

[brb33324-bib-0008] Fujioka, Y. , Hata, N. , Akagi, Y. , Kuga, D. , Hatae, R. , Sangatsuda, Y. , Michiwaki, Y. , Amemiya, T. , Takigawa, K. , Funakoshi, Y. , Sako, A. , Iwaki, T. , Iihara, K. , & Mizoguchi, M. (2021). Molecular diagnosis of diffuse glioma using a chip‐based digital PCR system to analyze IDH, TERT, and H3 mutations in the cerebrospinal fluid. Journal of Neuro‐Oncology, 152, 47–54. 10.1007/s11060-020-03682-7 33417137 PMC7910241

[brb33324-bib-0009] Gerardi, R. M. , Cannella, R. , Bonosi, L. , Vernuccio, F. , Ferini, G. , Viola, A. , Zagardo, V. , Buscemi, F. , Costanzo, R. , Porzio, M. , Giovannini, E. A. , Paolini, F. , Brunasso, L. , Giammalva, G. R. , Umana, G. E. , Scarpitta, A. , Iacopino, D. G. , & Maugeri, R. (2023). Forecasting molecular features in IDH‐wildtype gliomas: The state of the art of radiomics applied to neurosurgery. Cancers (Basel), 15, 940. 36765898 10.3390/cancers15030940 PMC9913449

[brb33324-bib-0010] Gihr, G. , Horvath‐Rizea, D. , Kohlhof‐Meinecke, P. , Ganslandt, O. , Henkes, H. , Härtig, W. , Donitza, A. , Skalej, M. , & Schob, S. (2022). Diffusion weighted imaging in gliomas: A histogram‐based approach for tumor characterization. Cancers (Basel), 14, 3393. 10.3390/cancers14143393 35884457 PMC9321540

[brb33324-bib-0011] Gihr, G. A. , Horvath‐Rizea, D. , Hekeler, E. , Ganslandt, O. , Henkes, H. , Hoffmann, K.‐T. , Scherlach, C. , & Schob, S. (2020). Histogram analysis of diffusion weighted imaging in low‐grade gliomas: In vivo characterization of tumor architecture and corresponding neuropathology. Frontiers in oncology, 10, 206. 10.3389/fonc.2020.00206 32158691 PMC7051987

[brb33324-bib-0012] Gonçalves, F. G. , Chawla, S. , & Mohan, S. (2020). Emerging MRI techniques to redefine treatment response in patients with glioblastoma. Journal of Magnetic Resonance Imaging, 52, 978–997. 10.1002/jmri.27105 32190946 PMC7492394

[brb33324-bib-0013] Gritsch, S. , Batchelor, T. T. , & Gonzalez Castro, L. N (2022). Diagnostic, therapeutic, and prognostic implications of the 2021 World Health Organization classification of tumors of the central nervous system. Cancer, 128, 47–58. 10.1002/cncr.33918 34633681

[brb33324-bib-0014] Hasanau, T. , Pisarev, E. , Kisil, O. , Nonoguchi, N. , Le Calvez‐Kelm, F. , & Zvereva, M. (2022). Detection of TERT promoter mutations as a prognostic biomarker in gliomas: Methodology, prospects, and advances. Biomedicines, 10, 728. 10.3390/biomedicines10030728 35327529 PMC8945783

[brb33324-bib-0015] Jiang, S. , Zou, T. , Eberhart, C. G. , Villalobos, M. A. V. , Heo, H.‐Y. , Zhang, Y. , Wang, Y. , Wang, X. , Yu, H. , Du, Y. , Van Zijl, P. C. M. , Wen, Z. , & Zhou, J. (2017). Predicting IDH mutation status in grade II gliomas using amide proton transfer‐weighted (APTw) MRI. Magnetic Resonance in Medicine, 78, 1100–1109. 10.1002/mrm.26820 28714279 PMC5561497

[brb33324-bib-0016] Jovčevska, I. (2018). Sequencing the next generation of glioblastomas. Critical Reviews in Clinical Laboratory Sciences, 55, 264–282. 10.1080/10408363.2018.1462759 29668342

[brb33324-bib-0017] Just, N. (2014). Improving tumour heterogeneity MRI assessment with histograms. British Journal of Cancer, 111, 2205–2213. 10.1038/bjc.2014.512 25268373 PMC4264439

[brb33324-bib-0018] Kanas, V. G. , Zacharaki, E. I. , Thomas, G. A. , Zinn, P. O. , Megalooikonomou, V. , & Colen, R. R. (2017). Learning MRI‐based classification models for MGMT methylation status prediction in glioblastoma. Computer Methods and Programs in Biomedicine, 140, 249–257. 10.1016/j.cmpb.2016.12.018 28254081

[brb33324-bib-0019] Kim, M. , Jung, S. Y. , Park, J. E. , Jo, Y. , Park, S. Y. , Nam, S. J. , Kim, J. H. , & Kim, H. S. (2020). Diffusion‐ and perfusion‐weighted MRI radiomics model may predict isocitrate dehydrogenase (IDH) mutation and tumor aggressiveness in diffuse lower grade glioma. European Radiology, 30, 2142–2151. 10.1007/s00330-019-06548-3 31828414

[brb33324-bib-0020] Li, G. , Li, L. , Li, Y. , Qian, Z. , Wu, F. , He, Y. , Jiang, H. , Li, R. , Wang, D. , Zhai, Y. , Wang, Z. , Jiang, T. , Zhang, J. , & Zhang, W. (2022). An MRI radiomics approach to predict survival and tumour‐infiltrating macrophages in gliomas. Brain, 145, 1151–1161. 10.1093/brain/awab340 35136934 PMC9050568

[brb33324-bib-0021] Li, Y. , Liu, Y. , Liang, Y. , Wei, R. , Zhang, W. , Yao, W. , Luo, S. , Pang, X. , Wang, Y. , Jiang, X. , Lai, S. , & Yang, R. (2022). Radiomics can differentiate high‐grade glioma from brain metastasis: A systematic review and meta‐analysis. European Radiology, 32, 8039–8051. 10.1007/s00330-022-08828-x 35587827

[brb33324-bib-0022] Malik, N. , Geraghty, B. , Dasgupta, A. , Maralani, P. J. , Sandhu, M. , Detsky, J. , Tseng, C.‐L. , Soliman, H. , Myrehaug, S. , Husain, Z. , Perry, J. , Lau, A. , Sahgal, A. , & Czarnota, G. J. (2021). MRI radiomics to differentiate between low grade glioma and glioblastoma peritumoral region. Journal of Neuro‐Oncology, 155, 181–191. 10.1007/s11060-021-03866-9 34694564

[brb33324-bib-0023] Nakamura, M. , Masutomi, K. , Kyo, S. , Hashimoto, M. , Maida, Y. , Kanaya, T. , Tanaka, M. , Hahn, W. C. , & Inoue, M. (2005). Efficient inhibition of human telomerase reverse transcriptase expression by RNA interference sensitizes cancer cells to ionizing radiation and chemotherapy. Human Gene Therapy, 16, 859–868. 10.1089/hum.2005.16.859 16000067

[brb33324-bib-0024] Park, C. J. , Han, K. , Kim, H. , Ahn, S. S. , Choi, D. , Park, Y. W. , Chang, J. H. , Kim, S. H. , Cha, S. , & Lee, S.‐K. (2021). MRI features may predict molecular features of glioblastoma in isocitrate dehydrogenase wild‐type lower‐grade gliomas. Ajnr American Journal of Neuroradiology, 42, 448–456. 10.3174/ajnr.A6983 33509914 PMC7959428

[brb33324-bib-0025] Peng, H. , Huo, J. , Li, B. , Cui, Y. , Zhang, H. , Zhang, L. , & Ma, L. (2021). Predicting iosocitrate dehydrogenase (IDH) mutation status in gliomas using multiparameter MRI radiomics features. Journal of Magnetic Resonance Imaging, 53, 1399–1407. 10.1002/jmri.27434 33179832

[brb33324-bib-0026] Rogers, A. W. , Vega‐Ramon, F. , Yan, J. , Del Río‐Chanona, E. A. , Jing, K. , & Zhang, D. (2022). A transfer learning approach for predictive modeling of bioprocesses using small data. Biotechnology and Bioengineering, 119, 411–422. 10.1002/bit.27980 34716712

[brb33324-bib-0027] Rohwer, N. , Zasada, C. , Kempa, S. , & Cramer, T. (2013). The growing complexity of HIF‐1alpha's role in tumorigenesis: DNA repair and beyond. Oncogene, 32, 3569–3576. 10.1038/onc.2012.510 23160373

[brb33324-bib-0028] Sekhar, A. , Biswas, S. , Hazra, R. , Sunaniya, A. K. , Mukherjee, A. , & Yang, L. (2022). Brain tumor classification using fine‐tuned GoogLeNet features and machine learning algorithms: IoMT enabled CAD system. IEEE Journal of Biomedical and Health Informatics, 26, 983–991. 10.1109/JBHI.2021.3100758 34324425

[brb33324-bib-0029] Śledzińska, P. , Bebyn, M. G. , Furtak, J. , Kowalewski, J. , & Lewandowska, M. A. (2021). Prognostic and predictive biomarkers in gliomas. International Journal of Molecular Sciences, 22, 10373. 10.3390/ijms221910373 34638714 PMC8508830

[brb33324-bib-0030] Stichel, D. , Ebrahimi, A. , Reuss, D. , Schrimpf, D. , Ono, T. , Shirahata, M. , Reifenberger, G. , Weller, M. , Hänggi, D. , Wick, W. , Herold‐Mende, C. , Westphal, M. , Brandner, S. , Pfister, S. M. , Capper, D. , Sahm, F. , & Von Deimling, A. (2018). Distribution of EGFR amplification, combined chromosome 7 gain and chromosome 10 loss, and TERT promoter mutation in brain tumors and their potential for the reclassification of IDHwt astrocytoma to glioblastoma. Acta Neuropathologica, 136, 793–803. 10.1007/s00401-018-1905-0 30187121

[brb33324-bib-0031] Parvaze, S. , Bhattacharjee, R. , Singh, A. , Ahlawat, S. , Patir, R. , Vaishya, S. , Shah, T. J. , & Gupta, R. K. (2023). Radiomics‐based evaluation and possible characterization of dynamic contrast enhanced (DCE) perfusion derived different sub‐regions of glioblastoma. European Journal of Radiology, 159, 110655. 10.1016/j.ejrad.2022.110655 36577183

[brb33324-bib-0032] Tian, H. , Wu, H. , Wu, G. , & Xu, G. (2020). Noninvasive prediction of TERT promoter mutations in high‐grade glioma by radiomics analysis based on multiparameter MRI. BioMed research international, 2020, 1. 10.1155/2020/3872314<./bib> PMC7245686 32509858

[brb33324-bib-0033] Wang, H. , Zhang, S. , Xing, X. , Yue, Q. , Feng, W. , Chen, S. , Zhang, J. , Xie, D. , Chen, N. , & Liu, Y. (2023). Radiomic study on preoperative multi‐modal magnetic resonance images identifies IDH‐mutant TERT promoter‐mutant gliomas. Cancer medicine, 12, 2524–2537. 10.1002/cam4.5097 36176070 PMC9939206

[brb33324-bib-0034] Wang, P. , Tang, Z. , Xiao, Z. , Hong, R. , Wang, R. , Wang, Y. , & Zhan, Y. (2022). Dual‐energy CT in differentiating benign sinonasal lesions from malignant ones: Comparison with simulated single‐energy CT, conventional MRI, and DWI. European Radiology, 32, 1095–1105. 10.1007/s00330-021-08159-3 34427744

[brb33324-bib-0035] Yamashita, K. , Hatae, R. , Hiwatashi, A. , Togao, O. , Kikuchi, K. , Momosaka, D. , Yamashita, Y. , Kuga, D. , Hata, N. , Yoshimoto, K. , Suzuki, S. O. , Iwaki, T. , Iihara, K. , & Honda, H. (2019). Predicting TERT promoter mutation using MR images in patients with wild‐type IDH1 glioblastoma. Diagnostic and Interventional Imaging, 100, 411–419. 10.1016/j.diii.2019.02.010 30948344

[brb33324-bib-0036] Yan, J. , Zhang, B. , Zhang, S. , Cheng, J. , Liu, X. , Wang, W. , Dong, Y. , Zhang, L. , Mo, X. , Chen, Q. , Fang, J. , Wang, F. , Tian, J. , Zhang, S. , & Zhang, Z. (2021). Quantitative MRI‐based radiomics for noninvasively predicting molecular subtypes and survival in glioma patients. NPJ Precision Oncology, 5, 72. 10.1038/s41698-021-00205-z 34312469 PMC8313682

[brb33324-bib-0037] Yang, K. , Wu, Z. , Zhang, H. , Zhang, N. , Wu, W. , Wang, Z. , Dai, Z. , Zhang, X. , Zhang, L. , Peng, Y. , Ye, W. , Zeng, W. , Liu, Z. , & Cheng, Q. (2022). Glioma targeted therapy: Insight into future of molecular approaches. Molecular Cancer, 21, 39. 10.1186/s12943-022-01513-z 35135556 PMC8822752

[brb33324-bib-0038] Zhang, H. , Zhang, H. , Zhang, Y. , Zhou , B. , Wu, L. , Lei, Y. , & Huang, B. (2023). Deep learning radiomics for the assessment of telomerase reverse transcriptase promoter mutation status in patients with glioblastoma using multiparametric MRI. Journal of Magnetic Resonance Imaging, 58(5), 1441–1451. 36896953 10.1002/jmri.28671

